# Molecular signature of adipose tissue in patients with both Non-Alcoholic Fatty Liver Disease (NAFLD) and Polycystic Ovarian Syndrome (PCOS)

**DOI:** 10.1186/1479-5876-11-133

**Published:** 2013-05-31

**Authors:** Ancha Baranova, Thuy Phuong Tran, Arian Afendy, Lei Wang, Amirhossein Shamsaddini, Rohini Mehta, Vikas Chandhoke, Aybike Birerdinc, Zobair M Younossi

**Affiliations:** 1 Betty and Guy Beatty Center for Integrated Research, Inova Health System, 3300 Gallows Road, Falls Church VA 22042, USA; 2 Center for the Study of Chronic Metabolic Diseases, School of System Biology, College of Science, George Mason University, Fairfax VA, USA; 3 Center for Liver Diseases, Inova Fairfax Hospital, Falls Church VA, USA

**Keywords:** NAFLD, PCOS, LDLR, M30, Apoptosis, Ninein, Resistin

## Abstract

**Background:**

Polycystic ovarian syndrome (PCOS) is one of the most common reproductive disorders with strong association with both insulin resistance and non-alcoholic fatty liver disease (NAFLD). To untangle the complex relationship between PCOS and NAFLD, we analyzed serum biomarkers of apoptosis, some adipokines and mRNA profiles in the visceral adipose tissue of obese patients with NAFLD who were also diagnosed with PCOS and compared to a group with NAFLD only.

**Methods:**

We included patients with biopsy-proven NAFLD and PCOS (N = 12) and BMI-matched biopsy-proven NAFLD patients without PCOS (N = 12). Expression levels of individual mRNAs and soluble serum biomarkers were compared by non-parametric Mann–Whitney test. The analysis also included Spearman rank correlation tests and multiple regression analysis. For co-correlated genes, the factor analysis was performed.

****Results**:**

The total serum levels of apoptotic biomarker M30 were significantly elevated in PCOS patients with liver steatosis as compared to non-PCOS NAFLD controls (P < 0.02), pointing that androgen-dependent proapoptotic PCOS environment that may directly contribute to NAFLD progression in these patients. Similarly, hyperandrogenism may explain the observed PCOS-specific decrease (P < 0.04) in adipose LDLR mRNA expression that may be connected to the proneness of PCOS patients to NAFLD. The levels of mRNA encoding angiogenesis-associated GSK-3B interacting protein ninein were also significantly increased in the adipose tissue of NAFLD patients with PCOS (P < 0.007). Furthermore, the levels of resistin positively correlated with expression levels of LDLR and prothrombin time (PT).

**Conclusion:**

An androgen-dependent proapoptotic PCOS environment may directly contribute to NAFLD progression in these patients. Hyperandrogenism may explain an observed decrease in adipose LDLR mRNA expression. An inflammation-associated increase in the release of resistin into circulation might contribute to the prothrombotic state observed under conditions associated with insulin resistance, including PCOS. The studies of larger cohorts of NAFLD with and without PCOS patients are needed to further assess these potential interactions.

## Background

Polycystic ovarian syndrome (PCOS) is one of the most common reproductive disorders with a strong association with insulin resistance [1]. The prevalence of PCOS in theCommunity setting varies from 5% to 10% of women of child-bearing age. The Rotterdam diagnostic criteria for PCOS include presence of any two of the following features: oligoovulation or anovulation, clinical or biochemical hyperandrogenism and radiologic documentation of polycystic ovaries. [2]. According to these criteria, the true prevalence rates for PCOS may be as high as 18% [3].

Importantly, PCOS is related not only to the alteration of the levels of hormones that regulate the normal development of eggs in the ovaries, but also to disturbances in other metabolic pathways, such as glucose and lipid metabolisms [4]. In fact, PCOS affects only 5% of lean women whereas it occurs in about 28% of obese women, indicating a relationship between PCOS and obesity [3,5]. In particular, obesity significantly worsens all metabolic and reproductive outcomes of patients with PCOS except for hirsutism [6].

Non-alcoholic fatty liver disease (NAFLD) is another manifestation of obesity and metabolic syndrome and is now recognized as a major cause of chronic liver disease, affecting 25% to 30% of the U.S. population [7]. Conversely, in obese and overweight individuals, NAFLD prevalence can be as high as 70% [7]. The pathological spectrum of NAFLD ranges from simple hepatic steatosis to more severe manifestations such as non-alcoholic steatohepatitis (NASH), hepatic fibrosis, and cirrhosis [8].

Both PCOS and NAFLD share crucial features of metabolic syndrome including visceral obesity, hypertension, dyslipidemia and insulin resistance [9,10]. Insulin resistance is observed in about 50% to 80% of PCOS patients [11] and in up to 80% of some groups of NAFLD patients [12]. Intuitively, one would assume insulin resistance to be always associated with obesity, but clinical evidence has revealed that it exists in both lean and obese women with PCOS [13,14]. As noted previously, insulin resistance is also a key event linking NAFLD to metabolic syndrome since clinical features of metabolic syndrome, such as type 2 diabetes, hypertension, dyslipidemia, and obesity, are commonly diagnosed in NAFLD patients [15]. Moreover, NAFLD is now considered to be the hepatic manifestation of metabolic syndrome [16] while PCOS may be the ovarian manifestation of metabolic syndrome [10].

Although both PCOS and NAFLD progress through the requisite steps of central obesity and insulin resistance, the two disorders are not always present in the same patient. Research on the commonalities of these two disorders has yielded conflicting conclusions, often confounded by the additional complications posed by the concurrent presence of metabolic syndrome. Despite these unresolved issues, it is evident that PCOS and NAFLD share some metabolic pathways influenced by both obesity and insulin resistance. Untangling this complex relationship may provide better options for the treatment of these diseases, as well as the prevention of associated risk factors.

An aberrant adipokine production in visceral adipose tissue has been implicated in both metabolic syndrome and the pathogenesis of NAFLD/NASH [17,18]. So far, however, there is a paucity of information on PCOS related gene expression patterns in the adipose tissue. In this study, our aim was to analyze the mRNA profiles in the visceral adipose tissue of obese patients with biopsy-proven NAFLD with and without PCOS. To further support our findings, we augmented our data by protein expression studies using serum samples collected from the same patients at the time of liver biopsy and adipose tissue sample collection.

## Methods

### Sample collection, storage and selection

A study set of 24 visceral adipose tissues (VAT) accompanied by serum samples was selected from existing biobank of more than 400 archived specimens previously collected from obese patients at the time of their bariatric surgeries after informed consent and immediately frozen with liquid nitrogen before storage at −80 C. Thorough histories were obtained including alcohol use, history of diabetes mellitus, hypertension, or hyperlipidemia (all were defined by clinical diagnosis requiring medical therapy). Height, weight, hip, and waist measurements were collected pre-surgery along with laboratory tests included fasting glucose, serum aminotransferases (ALT and AST) and lipid panel. Patients with evidence of excessive alcohol use (>10 g/d), other causes of liver disease (e.g., viral hepatitis, autoimmune liver disease, iron overload) and those receiving treatment with PPAR-γ agonists were not included in this analysis. At the time of surgery, each patient underwent a liver biopsy which was reviewed by the study pathologist. In all cases, histological assessment of liver biopsies had excluded NASH, thus the extent of NAFLD in all cases was limited to steatosis of the liver. Diagnosis of PCOS was established according to Rotterdam diagnosis criteria and a combination of laboratory tests. Non-PCOS causes of anovulation and infertility were ruled out. In entire database, a total of 12 records satisfied both non-NASH NAFLD and PCOS criteria outlined above. These 12 records were further matched for age, BMI, AST and ALT levels as well a number of other parameters with non-NASH NAFLD, non-PCOS subjects form the same database. The ELISAs and qRT-PCR assays were performed after initial selection of the patients and matching. The study was approved by the Institutional Review Board of Inova Fairfax Hospital (Federal Assurance FWA00000573).

### MRNA extraction

The total mRNAwas extracted from ~100 mg of frozen adipose tissue samples using Aurum™ Total RNA Fatty Acid and Fibrous Tissue Kit (Bio-Rad, Hercules, California) as suggested by manufacturer. The quality of the extracted mRNA was assessed spectrophotometrically using the A260/A280 ratio that in all samples was between 1.8 and 2.0. In addition, the presence of intact RNA was also confirmed electrophoretically at 1% agarose gels with 28S and 18S rRNA bands clearly visible and sharp.

### First strand CDNA synthesis

Freshly extracted RNA samples were processed to synthesize CDNA using RT^2^ First Strand Kit (Qiagen, Valencia, CA). Reverse transcription reactions were performed using approximately 1.5 μg of total RNA. Reactions were heated at 42°C for 5 min in a total volume of 10.0 μl in the presence of 2.0 μl of 5X gDNA Elimination Buffer and chilled on ice immediately for at least 1 min. Then, a RT cocktail consisting of 4 μl of PC3 (5X first strand buffer), 2 μl of RE3 (RT enzyme mix), 1 μl P2 (Primer and External Control Mix) and 3 μl nuclease-free H_2_O were added. The mixture was then incubated at 42C for exactly 15 min and then immediately stopped by heating to 95 C for 5 minutes. 91 μl of H_2_O was added to each 20-μl ofCDNA synthesis reaction. All extracted RNAs and subsequent CDNA samples were stored in −20 C short-term and at −80 C long-term.

### Real- Time Reverse Transcription Polymerase Chain Reaction (qRT-PCR)

Target genes for this study were selected among regulatory genes directly or indirectly involved in the insulin signaling. Hence, the candidate genes were picked from the carbohydrate metabolism pathway including: fructose-1,6-bisphosphatase 1 (*FBP1*), glycogen synthase kinase-3β interacting protein ninein (*NIN*) and protein phosphatase 1 (*PPP1CA*); from the lipid metabolism pathway: low density lipoprotein receptor (*LDLR*), sterol regulatory element binding transcription factor 1 (*SREBF1*), acetyl-CoACarboxylase alpha (*ACACA*), sorbin and SH3 domain containing 1 (*SORBS1*), RAF proto-oncogene serine/threonine-protein kinase (*RAF1*); and target genes for peroxisome proliferator-activated receptor subfamily of nuclear receptors, such as acyl-CoA oxidase 1 (*ACOX1*) and peroxisome proliferator-activated receptor gamma (*PPARG*). For endogenous reference gene, we used RNA polymerase II (*RPII*) that was validated in in previously published study [19]. RPII was profiled simultaneously with each qRT-PCR reaction.

PCR primers for *ACTB, B2M, FBP1, NIN, PPP1CA, LDLR, SREBF1, ACACA, RAF1, ACOX1, SORBS1* and *PPARG* were purchased at Real Time Primers, USA. Primers for the *RPII* and *SORBS1* were custom designed using Oligo Perfect Designer software (Invitrogen, USA) and synthesized at Invitrogen, USA. Gene functions and primer sequences are summarized in Table 1.

**Table 1:** Sequences of primers used in qRT-PCR

**Gene name**	**Forward (F)**	**Functions**
	**Reverse (R)**	
Acetyl-CoACarboxylase Alpha (*ACACA*)	5′-CCCAGATTCTGCGTT TAAGA -3′	Long-chain fatty acid synthesis
	5′-CATCCACATGT AAGCACCA -3′	
Sterol Regulatory Element Binding Transcription Factor 1 (*SREBF1*)	5′-TACATTCGCTTTCTGCAACA -3′	Transcriptional activator for lipid homeostasis
	5′- GTCAGGTTCC TCCACCTC -3′	
Peroxisome Proliferator-Activated Receptor Gamma (*PPARG*)	5′-CCC AAG TTT GAG TTT GCT GT -3′	Transcription factor
	5′- AAC AGC TGT GAG GAC TCA GG3′	
Glycogen Synthase Kinase 3 Beta interaction protein ninein (*NIN*)	5′- GAA TGT GGA TGG AGA GAT GC	Glycogen metabolism
	5′- ATT GCT GAT GAG GTT GTG GT 3′	
RAF proto-oncogene serine/threonine-protein kinase (*RAF1*)	5′- ATCCGA ATGCAG GAT AAC AA 3′	Regulator inCell fate decisions
	5′- AAG ATC TGG GGA GGC ATA TC3′	
Fructose-1, 6-Bisphosphatase 1 (*FBP1*)	5′- TCA ACT GCT TCA TGC TGG AC -3′	Gluconeogenesis regulator
	5′-CGT AGACCA GAG TGC GAT GA3′	
Acyl-CoA Oxidase 1 (*ACOX1*)	5′-CTG AAG GCT TTC ACC TCC TG -3′	Fatty acid Beta-Oxidation pathway
	5′-CAT GCC ACACACCAACTT TC -3′	
Low Density Lipoprotein Receptor (*LDLR*)	5′-CCA AACCCC TAA ACTCAG GA 3′	Receptor-mediated endocytosis of LDL
	5′- AAG TGGCATCAT TTG GTG AA 3′	
Sorbin and SH3 DomainContaining 1 (*SORBS1*)	5′-CTGCAAGCCCACAGTTTTCCAGT-3′	Insulin-stimulated glucose transport
	5′-CGAGCAGCTTTCCTCCCCGC-3′	
Protein phosphatase 1 (*PPP1CA*)	5′- ACC TGC AGTCTA TGG AGC AG 3′	Glycogen metabolism
	5′- TAGCCG TCT TCT ACC ACC TG -3′	

Gene expression levels were quantified by qRT-PCR using the gene-specific primers with individual Tm’s between 58 C and 60 C. Primer specificity was validated by both melting curve analysis and gel electrophoresis. The real-time PCR mixtures containing 1 μl of the RT sample, 250 nM each of forward and reverse primers (Quiagen, Valencia, CA) and 2X SsoFast™ EvaGreen® Supermix (Bio-Rad Laboratories, Hercules, CA) were carried out in a total volume of 10 μl. Reactions were performed in a 96-well format in the BioRad CFX96 Real Time System (BioRad Laboratories, Hercules, CA). After melting curve based quality control, the RT-PCR data were transformed using the ΔCt method.

### Enzyme-linked immunosorbent assay (ELISA)

Previously collected and frozen serum samples were used to determine levels of the cytokines of interest by 96-well sandwich ELISA kits (Total CK18 and Caspase Cleaved CK 18 Prototype - Peviva, Bromma, Sweden; Human Adiponectin and Human Resistin – R&D Systems, Minneapolis, MN; Insulin EIA – Alpco Diagnosis, Salem, NH). The detection limits forCK18 kits were: for Total CK18 at 11 U/L, for caspase- cleaved CK18 at 5 U/L. For adipokine assays, the sensitivity limits were at 0.891 μg/mL for the adiponectin, at 0.055 ng/mL for the resistin and at 0.399 μIU/mL for the insulin, as reported by manufacturers. The optical densities (ODs) were measured using the ELx800 spectrophotometer at 450 nm with a reference wavelength of 620 nm.

### Data analysis

To compare the expression levels of individual mRNAs, the statistical analysis by non-parametric Mann–Whitney test was performed. The analysis also included Spearman rank correlation tests and multiple regression analysis (MATLAB®, The MathWorks, Inc. Natick, MA). P values < 0.05 were considered significant. The multiple test corrections were carried out using Benjamini-Hochberg-Yekutieli procedure that controls the false discovery rate under positive dependence assumptions reflecting known phenomenon of co-correlation of expression levels for genes involved in the same cellular or organismal process. The procedure was executed using Bioconductor. Only comparisons that yielded significant findings (after correction) were reported.

For co-correlated genes (*ACACA, SREBF1, FBP1, PPP1CA, ACOX1* and *PPARG*), the factor analysis was performed with default rotation parameter and specified single factor output. The estimates of factor loadings were based on data from all subjects. The number of factors was estimated using the maximum likelihood method. The matrices of factor loadings were rotated by orthogonal transformation to simplify the resulting structure and ensure the independency of the factors. The common factor obtained through this approach was subject to a univariate correlation analyses with glucose level, insulin level, individual adipokine levels and HOMA scores.

## Results

### Clinical and biochemical characteristics of biopsy-proven NAFLD patients with and without PCOS

For this study, we selected a set of biopsy-proven NAFLD patients (N = 24) with (N = 12) or without (N = 12) PCOS. In all cases, histological assessment of liver biopsies had excluded NASH, thus the extent of NAFLD in all cases was limited to steatosis of the liver. NAFLD patients with PCOS and without PCOS were matched for age, BMI, AST and ALT levels as well a number of other parameters (Table 2). Diagnosis of PCOS was established according to Rotterdam diagnosis criteria and a combination of laboratory tests. Non-PCOS causes of anovulation and infertility were ruled out. The clinical and biochemical descriptions are provided in Table 2.

**Table 2:** **Clinical and biochemical** c**haracteristics of PCOS-NAFLD patients and** c**ontrols**

	**Non-PCOS (n = 12)**	**PCOS (n = 12)**	**p-value**
Age, years	37.6 +/− 10.0	35.2 +/− 9.60	NS
Heights, cm	165.0 +/− 11.0	168.1 +/− 3.9	NS
Weight, kg	127.4 +/− 11.6	138.7 +/− 39.4	NS
BMI (kg/cm2)	44.1 +/− 3.9	45.0 +/− 3.6	NS
AST (U/L)	23.5 +/− 15.00	23.3 +/− 10.4	NS
ALT (U/L)	28.7 +/− 21.9	29.9 +/−15.3	NS
AST/ALT	0.96 +/− 0.35	0.85 +/− 0.24	NS
TCHOL (mg/dL)	187.3 +/− 31.2	178.5 +/− 34.9	NS
LDL (mg/dL)	101.4 +/− 27.5	108.1 +/−37.3	NS
HDL (mg/dL)	43.6 +/− 11.1	54.2 +/−14.6	NS
TRIG (mg/dL)	177.1 +/−110.52	133.7 +/−34.8	NS

### Gene expression levels of glycogen synthase kinase 3β interacting protein ninein (NIN) and Low-density lipoprotein receptor (LDLR) are altered in NAFLD + PCOS group

To identify specificComponents of the insulin signaling pathway that could potentially be involved in the pathogenesis of PCOS in NAFLD patients, the expression levels of ten target genes related to insulin regulation were investigated: *FBP1, NIN, PPP1CA, LDLR, SREBF1, ACACA, RAF1, ACOX1, SORBS1* and *PPARG*.

**Figure 1:**
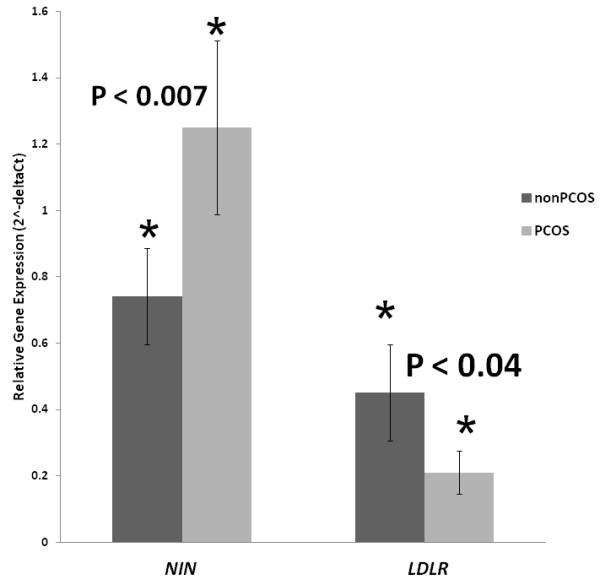
**Differential gene expression of *NIN *and *LDLR *genes in PCOS and non-PCOS.** (Fold change: 1.65, p = 0.009 and 0.51, p = 0.042, respectively).

Among these genes, expression levels of *NIN* and *LDLR* mRNAs were significantly altered in the PCOS NAFLD group as compared to the non-PCOS NAFLD group (upregulated 1.65 fold with p < 0.009 and downregulated 0.51 fold with p < 0.04, respectively) (Figure 1). The gene expression levels for the rest of the target genes were not significantly different between groups of patients (Table 3).

**Table 3:** Adipokine and gene expression levels in PCOS-NAFLD group and non-PCOS NAFLD

	**Non-PCOS (n = 12)**	**PCOS (n = 12)**	**P value**
M65, U/L	284.18 +/− 73.81	299.92 +/− 98.87	NS
M30, U/L	119.07 +/− 7.94	224.93 +/− 111.87	< 0.03
Adiponectin, μg/ml	6.88 +/− 4.50	6.98 +/− 2.34	NS
Resistin, ng/ml	14.25 +/− 5.50	11.17 +/− 4.17	NS
Insulin (MicroIU/ml)	16.9 +/− 11.9	21.7 +/− 23.7	NS
ACACA	0.32 +/− 0.22	0.30 +/− 0.17	NS
SREBF1	0.56 +/− 0.43	0.67 +/− 0.35	NS
NIN	0.74 +/− 0.37	1.25 +/− 0.64	< 0.009
SORBS1	0.00173 +/− 0.00101	0.00216 +/− 0.00124	NS
FBP1	0.77 +/− 0.46	0.55 +/− 0.35	NS
PPP1CA	5.61 +/− 3.05	5.37 +/− 1.88	NS
RAF1	1.58 +/− 0.84	1.55 +/− 0.73	NS
ACOX1	3.82 +/− 3.91	3.66 +/− 3.27	NS
PPARG	8.96 +/− 8.29	10.56 +/− 10.49	NS
LDLR	0.45 +/− 0.38	0.21 +/− 0.16	< 0.042

### Caspase cleaved CK18 levels are elevated significantly in PCOS group

**Figure 2:**
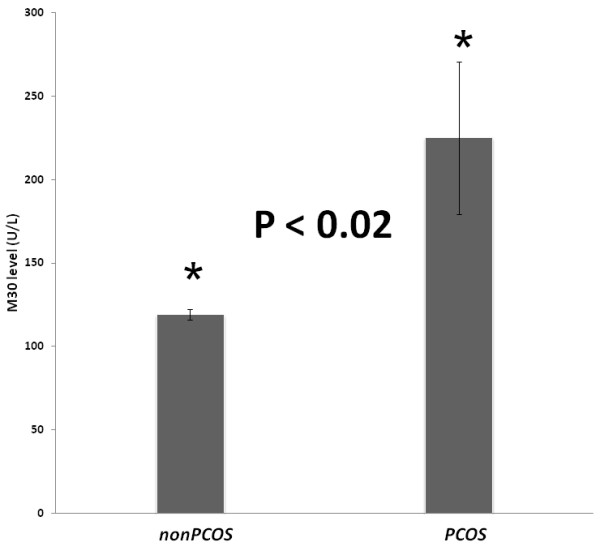
Caspase-cleavedCK18 (M30) levels in PCOS and non-PCOS groups.

Since cell death is implicated in the pathogenesis of both PCOS and NAFLD, we also compared the serum biomarkers of cell death in both PCOS and non-PCOS NAFLD patients. The serum total cytokeratin 18 (M65) levels reflect the amount of total epithelial cell death, regardless of the cause of death, the caspase cleaved cytokeratin 18 (M30) is a biomarker of apoptosis. The serum M65 levels in the PCOS NAFLD and the non-PCOS-NAFLD groups were similar (Table 4), while the levels of M30 were higher in PCOS–NAFLD group as compared to that in the non PCOS-NAFLD group (224.93 U/L +/− 111.87 U/L vs. 119.07 U/L +/− 7.94 U/L, P < 0.03) (Table 3 and Figure 2).

**Table 4:** **Spearman** c**orrelation** c**oefficients (r) with p < 0.05**

**Correlating pair of the variables**	**Correlation Coefficient (r)**	**P value**
Resistin - HOMA	0.66	< 0.0006
Resistin - Insulin	0.67	< 0.00005
Resistin - PT	0.42	< 0.045
LDLR - Resistin	0.721	< 0.0325
LDLR – PT	0.89	< 0.0076

### Resistin levels correlate with expression of LDLR and measures of insulin resistance

The serum levels of resistin, adiponectin, and insulin were not significantly different between NAFLD patients with or without PCOS (Table 3). The levels of resistin were positively correlated with expression levels of *LDLR*, prothrombin time, serum levels of insulin and HOMA scores. Additionally, levels of *LDLR* mRNA were also correlated with prothrombin time (Table 4).

### Levels of mRNAs encoding proteins involved in glucose metabolism and lipid metabolism correlate with each other

PPARG and ACOX1 proteins regulate of adipocyte differentiation. Both of these proteins have been implicated in both obesity and type 2 diabetes. In our study, expression levels of *PPARG* mRNA showed significant positive correlation with levels of mRNAs encoded by genes involved in fatty acid metabolism (*ACACA*, r = 0.742, p < 0.014), cholesterol metabolism (*SREBF1*, r = 0.830, p < 0.006), glucose metabolism (*FBP1*, r = 0.883, p < 0.003) and glycogen metabolism (*PPP1CA*, r = 0.700, p < 0.043 and *ACOX1*, r < 0.903, p = 0.001). Expression levels for mRNAs of *ACOX1, SREBF1, FBP1, ACACA* and *PPP1CA* genes wereCo-correlated (Table 4). This co-correlation was observed in both PCOS and non-PCOS groups and was confirmed by factor analysis, where p-values for co-correlation to common factor varied from < 0.3 to < 1.56e^-5^. None of the other measured parameters, including glucose, insulin, adipokine levels and HOMA scores, were co-correlated to the expression levels for this group of genes.

## Discussion

Recent studies have revealed that both PCOS and NAFLD are strongly associated with insulin resistance, metabolic syndrome and obesity [10]. This is especially true for women with visceral obesity [3,4]. As women with PCOS may be at increased risk for developing NAFLD and conversely, women with NAFLD may be at risk for PCOS, the screenings for these chronic and inter-related diseases have been proposed in the respective cohorts [10]. Given the overlap in the pathogenesis, molecular pathways involved in the pathogenesis of these two different manifestations of metabolic syndrome will be of great interest.

In our previous study, we have shown that despite similar clinical and laboratory profiles to the obese controls, PCOS patients seem to be at increased risk for histologic NASH [20]. In this study we specifically selected the population of patients with both PCOS and NAFLD that have not yet progressed to NASH according to their liver biopsies (N = 12) and BMI-matched this population with non-PCOS non-NASH NAFLD controls (N = 12). In fact, our results show that levels of adiponectin, resistin, insulin and HOMA scores as well as number of other clinical and biochemical parameters were not significantly different between these two groups of patients (Table 2).

Nevertheless, we were able to show that level of caspase-cleaved CK18 (M30) was significantly elevated in PCOS patients with NAFLD, suggesting a more prominent role for apoptosis in these patients. In fact, this data indicate that presence of PCOS may indicate a “more intense pro-apoptotic” environment. This observation confirms findings of Tan et al. who compared serum apoptosis biomarkers in 186 PCOS patients and 73 age-matched controls, and found that M30 levels are higher in PCOS patients than in normal controls even after correction for BMI and suggested that NASH should be prevalent in PCOS cohorts [21]. However, our study takes this investigation one step further, as its design specifically excluded patients with histologically confirmed NASH, thus indicating that an increase in apoptosis is an early feature of NAFLD observed in PCOS subjects, or that an overall increase in apoptosis is a general feature of PCOS. One of the most prominent features of PCOS is a hyperproduction of androgens, like testosterone, dihydrotestosterone (DHT) and dehydroepiandrosterone (DHEA). Androgens are known as proapotototic agents and were shown to act upon many types of peripheral cells, including hepatocytes [22]. For example, DHT administration results in apoptosis of androgen-sensitive liver cells, that is, at least in part, realized through PKR/eIF2α/GADD153 cascades [22]. It is possible that the patients with concomitant NAFLD are more prone to the development of NASH, as androgen-instigated apoptotic processes in their livers actively contribute to the progression of NAFLD. To further investigate this hypothesis, the study of larger cohorts of PCOS patients are needed, including subgroups without any histological sign of the liver disease and those without an excess of androgens.

Our present study also provides interesting insights into the PCOS-associated changes in the expression levels of genes involved in specific aspects of the lipid metabolism and theCarbohydrate metabolism in adipose. Importantly, in adipose tissue specimen collected from PCOS patients, decreased mRNA levels for LDL receptor (*LDLR*) were observed. The expression of *LDLR* gene is a subject of estrogen control that is maintained through the estrogen-responsive region adjacent to the sterol response element within the LDLR promoter. In addition, in hepatocytes, androgen receptor agonists were shown to attenuate the estrogen-induced up-regulation of *LDLR*[23]. It seems that hyperandrogenic phenotype of PCOS may have a direct suppressive influence on LDLR receptor levels both in adipocytes and in the liver. As the LDL receptor plays a major role in the clearance of apoB and apoE-containing lipoproteins, its downregulation should prolong the plasma half-life of VLDL and LDL and, therefore, have steatogenic effects. In accordance with this logic, *Ldlr−/−* mice have increased sensitivity for oxLDL-induced inflammation, apoptosis and fibrosis [24]. On diabetogenic, cholesterol-augmented diet, *Ldlr−/c* mice develop a distinct hepatic phenotype characterized by increased inflammation and oxidative stress [25]. Thus, an observed decrease in LDLR mRNA in PCOS might be connected to the proneness of PCOS patients with concomitant liver disease to the progression to NASH.

The only other mRNA that was differentially expressed in PCOS adipose tissue was one encoding for centrosomal protein ninein (*NIN*). NIN was selected for this study as it interacts with kinase GSK3beta that is hyperactivated and resistant to down-regulation by insulin in adipocytes of women with PCOS [26]. As of now, the connection of the ninein to PCOS phenotype is unclear. One possible clue that can make this connection is that the ninein is critical for the formation of vascular tubes [27]. An observed increase in the expression of ninein aligns well with hypervascularity of the ovarian theca interna and stroma commonly observed in patients with PCOS [28].

When PCOS and non-PCOS NAFLD groups were compared, no difference in the levels of resistin, adiponectin and insulin were observed, thus, confirming the findings of others [29,30] and pointing to the fact that the levels of these hormones are defined by underlying metabolic states and patient’s BMI, rather than by PCOS. On the other hand, when the entire group of NAFLD patients was analyzed together, the levels of *LDLR* mRNA expression in adipose were positively correlated with serum levels of resistin. This is an important observation as resistin was recently shown to downregulate LDLR at the protein level *in vitro*, both in HepG2 and in primary hepatocytes, through an increase in cellular expression of the recently identified protease, PCSK9, which enhances intracellular LDLR lysosomal degradation [31]. Thus, our study indicates that the same phenomenon may take place *in vivo*, and highlighs resistin as a possible therapeutic target to be manipulated in patients with elevated serum LDL levels.

Additionally, both LDLR mRNA expression and resistin levels were correlated with the measure of the extrinsic pathway of coagulation, prothrombin time (PT). An increase in PT is common in insulin resistance and in PCOS that are both regarded as low-grade systemic coagulation conditions [32,33]. Recenty, resistin was shown to induce a procoagulant state in HUVECs by inducing both the expression of tissue factor (TF) and the activity of factor Xa [34]. Correlative observations made in metabolic syndrome cohorts showed that resistin is strongly associated with hypercoagulative and hypofibrinolitic activities [35]. It seems that inflammation-associated increases in the release of resistin into circulation might contribute to the prothrombotic state observed under diabetic conditions.

The limitation of our study, as with most molecular studies, is small sample size, which may contribute to the lack of statistically significant differences of group comparison analysis of changes in gene expressions as well as limit our analysis of correlations. However, in our design we attempted the mitigation of these limitations by matching the PCOS and non-PCOS groups of study subjects by age, BMI and liver enzyme as well as the histology of their liver. We hope that the findings of our study will aids in untangling complex relationship between NAFLD and PCOS, and point toward novel avenues for similarly designed studies in larger cohorts.

## Conclusion

In conclusion, we report that the total serum levels of apoptotic biomarker M30 are significantly elevated in PCOS patients with liver steatosis as compared to non-PCOS NAFLD controls, suggesting that an androgen-dependent proapoptotic PCOS environment may directly contribute to NAFLD progression in these patients. Similarly, hyperandrogenism may explain an observed decrease in adipose LDLR mRNA expression that may be connected to the proneness of PCOS patients with concomitant liver disease to the progression to NASH. Additionally, our study suggests that inflammation-associated increase in the release of resistin into circulation might contribute to the prothrombotic state observed under conditions associated with insulin resistance, including PCOS.

## Competing interests

The authors declare that they have no competing interests.

## Authors’ contributions

ABar, ABir, VC and ZY designed the study. AA collected the samples. TPT performed qRT-PCR and ELISAs. LW, AS and RM performed statistical analysis. ABar, TPT and ABir wrote the manuscript. All authors read and approved the final manuscript.

## Authors’ information

ABar is an Associate Professor at the School of Systems Biology, College of Science, George Mason University (SSBCOS GMU). RM and LW are PhD students in the School of SSBCOS GMU. TPT is a student in MS in Biosciences program at SSBCOS GMU. VC is a Dean of College of Science, George Mason University. AS is a student in MS in Bioinformatics program at SSBCOS GMU. VC is a Dean of College of Science, George Mason University. ABir is Research Assistant Professor at SSBCOS GMU. AA is a Research Associates at Betty and Guy Beatty Center for Integrated Research, Inova Health System. ZY is a chairman, Department of Medicine, Inova Fairfax Hospital and Vice President for Research, Inova Health System.

## References

[B1] Diamanti-Kandarakis E Papavassiliou AG Molecular mechanisms of insulin resistance in polycystic ovary syndrome Trends Mol Med 2006 12 324 332 10.1016/j.molmed.2006.05.006 16769248

[B2] Rotterdam ESHRE/ASRM-Sponsored PCOS consensus workshop group Revised 2003 consensus on diagnostic criteria and long-term health risks related to polycystic ovary syndrome (PCOS) Hum Reprod 2004 19 41 47 14688154 10.1093/humrep/deh098

[B3] Alvarez-Blasco F Botella-Carretero JI San Millan JL Escobar-Morreale HF Prevalence and characteristics of the polycystic ovary syndrome in over-weight and obese women Arch Intern Med 2006 166 2081 2086 10.1001/archinte.166.19.2081 17060537

[B4] Teede H Deeks A Moran L Polycystic ovary syndrome: a complex condition with psychological, reproductive and metabolic manifestations that impacts on health across the lifespan BMC Med 2010 8 41 10.1186/1741-7015-8-41 20591140 PMC2909929

[B5] Bjorntorp P Metabolic implications of body fat distribution Diab Care 1991 14 1132 1143 10.2337/diacare.14.12.1132 1773700

[B6] Lim SS Norman RJ Davies MJ Moran LJ The effect of obesity on polycystic ovary syndrome: a systematic review and meta-analysis Obes Rev 2013 14 95 109 10.1111/j.1467-789X.2012.01053.x 23114091

[B7] Vernon G Baranova A Younossi ZM Systematic review: the epidemiology and natural history of non-alcoholic fatty liver disease and non-alcoholic steatohepatitis in adults Aliment Pharmacol Ther 2011 34 274 285 10.1111/j.1365-2036.2011.04724.x 21623852

[B8] Younossi ZM Venkatesan C A 2012 clinical update for internists in adult nonalcoholic fatty liver disease Panminerva Med 2012 54 29 37 22278114

[B9] Brzozowska MM Ostapowicz G Weltman MD An association between non-alcoholic fatty liver disease and polycystic ovarian syndrome J Gastroenterol Hepatol 2009 24 243 247 10.1111/j.1440-1746.2008.05740.x 19215335

[B10] Baranova A Tran TP Birerdinc A Younossi ZM Systematic review: association of polycystic ovary syndrome with metabolic syndrome and non-alcoholic fatty liver disease Aliment Pharmacol Ther 2011 33 801 814 10.1111/j.1365-2036.2011.04579.x 21251033

[B11] Legro RS Castracane VD Kauffman RP Detecting insulin resistance in polycystic ovary syndrome: purposes and pitfalls Obstet Gynecol Surv 2004 59 141 154 10.1097/01.OGX.0000109523.25076.E2 14752302

[B12] Ciba I Widhalm K The association between non-alcoholic fatty liver disease and insulin resistance in 20 obese children and adolescents Acta Paediatr 2007 96 109 112 17187615 10.1111/j.1651-2227.2007.00031.x

[B13] Stepto NK Cassar S Joham AE Hutchison SK Harrison CL Goldstein RF Teede HJ Women with polycystic ovary syndrome have intrinsic insulin resistance on euglycaemic-hyperinsulaemic clamp Hum Reprod 2013 28 777 784 10.1093/humrep/des463 23315061

[B14] Diamanti-Kandarakis E Dunaif A Insulin resistance and the polycystic ovary syndrome revisited: an update on mechanisms and implications Endocr Rev 2012 33 981 1030 10.1210/er.2011-1034 23065822 PMC5393155

[B15] Chitturi S Farrell GC Etiopathogenesis of nonalcoholic steatohepatitis Semin Liver Dis 2001 21 27 41 10.1055/s-2001-12927 11296694

[B16] Kim CH Younossi ZM Nonalcoholic fatty liver disease: a manifestation of the metabolic syndrome CleveClin J Med 2008 75 721 728 10.3949/ccjm.75.10.721 18939388

[B17] Estep JM Baranova A Hossain N Elariny H Ankrah K Afendy A Chandhoke V Younossi ZM Expression of cytokine signaling genes in morbidly obese patients with non-alcoholic steatohepatitis and hepatic fibrosis Obes Surg 2009 19 617 624 10.1007/s11695-009-9814-x 19280268

[B18] Baranova A Randhawa M Jarrar M Younossi ZM Adipokines and melanocortins in the hepatic manifestation of metabolic syndrome: nonalcoholic fatty liver disease Expert Rev Mol Diagn 2007 7 195 205 10.1586/14737159.7.2.195 17331066

[B19] Mehta R Birerdinc A Hossain N Afendy A Chandhoke V Younossi Z Baranova A Validation of endogenous reference genes for qRT-PCR analysis of human visceral adipose samples BMC Mol Biol 2010 11 39 10.1186/1471-2199-11-39 20492695 PMC2886049

[B20] Hossain N Stepanova M Afendy A Nader F Younossi Y Rafiq N Goodman Z Younossi ZM Non-alcoholic steatohepatitis (NASH) in patients with polycystic ovarian syndrome (PCOS) Scand J Gastroenterol 2011 46 479 484 21114431 10.3109/00365521.2010.539251

[B21] Tan S Bechmann LP Benson S Dietz T Eichner S Hahn S Janssen OE Lahner H Gerken G Mann K Canbay A Apoptotic markers indicate nonalcoholic steatohepatitis in polycystic ovary syndrome J Clin Endocrinol Metab 2010 95 343 348 10.1210/jc.2009-1834 19906783

[B22] Dai R Yan D Li J Chen S Liu Y Chen R Duan C Wei M Li H He T Activation of PKR/eIF2α signaling cascade is associated with dihydrotestosterone-induced cell cycle arrest and apoptosis in human liver cells J Cell Biochem 2012 113 1800 1808 22228470 10.1002/jcb.24051

[B23] Croston GE Milan LB Marschke KB Reichman M Briggs MR Androgen receptor-mediated antagonism of estrogen-dependent low density lipoprotein receptor transcription in cultured hepatocytes Endocrinology 1997 138 3779 3786 10.1210/en.138.9.3779 9275065

[B24] Bieghs V Van Gorp PJ Wouters K Hendrikx T Gijbels MJ van Bilsen M Bakker J Binder CJ Lütjohann D Staels B Hofker MH Shiri-Sverdlov R LDL receptor knock-out mice are a physiological model particularly vulnerable to study the onset of inflammation in non-alcoholic fatty liver disease PLoS One 2012 7 e30668 10.1371/journal.pone.0030668 22295101 PMC3266276

[B25] Subramanian S Goodspeed L Wang S Kim J Zeng L Ioannou GN Haigh WG Yeh MM Kowdley KV O’Brien KD Pennathur S Chait A Dietary cholesterol exacerbates hepatic steatosis and inflammation in obese LDL receptor-deficient mice J Lipid Res 2011 52 1626 1635 10.1194/jlr.M016246 21690266 PMC3151683

[B26] Chang W Goodarzi MO Williams H Magoffin DA Pall M Azziz R Adipocytes from women with polycystic ovary syndrome demonstrate altered phosphorylation and activity of glycogen synthase kinase 3 Fertil Steril 2008 90 2291 2297 10.1016/j.fertnstert.2007.10.025 18178198 PMC2744855

[B27] Matsumoto T Schiller P Dieterich LC Bahram F Iribe Y Hellman U Wikner C Chan G Claesson-Welsh L Dimberg A Ninein is expressed in the cytoplasm of angiogenic tip-cells and regulates tubular morphogenesis of endothelial cells Arterioscler Thromb Vasc Biol 2008 28 2123 2130 10.1161/ATVBAHA.108.169128 18772498

[B28] Ferrara N Frantz G LeCouter J Dillard-Telm L Pham T Draksharapu A Giordano T Peale F Differential expression of the angiogenic factor genes vascular endothelial growth factor (VEGF) and endocrine gland-derived VEGF in normal and polycystic human ovaries Am J Pathol 2003 162 1881 1893 10.1016/S0002-9440(10)64322-2 12759245 PMC1868136

[B29] Toulis KA Goulis DG Farmakiotis D Georgopoulos NA Katsikis I Tarlatzis BC Papadimas I Panidis D Adiponectin levels in women with polycystic ovary syndrome: a systematic review and a meta-analysis Hum Reprod Update 2009 15 297 307 10.1093/humupd/dmp006 19261627

[B30] Barber TM Franks S Adipocyte biology in polycystic ovary syndrome Mol Cell Endocrinol 2012 373 68 76 23084978 10.1016/j.mce.2012.10.010

[B31] Melone M Wilsie L Palyha O Strack A Rashid S Discovery of a new role of human resistin in hepatocyte low-density lipoprotein receptor suppression mediated in part by proprotein convertase subtilisin/kexin type 9 J Am Coll Cardiol 2012 59 1697 1705 10.1016/j.jacc.2011.11.064 22554600

[B32] Kebapcilar L Taner CE Kebapcilar AG Sari I High mean platelet volume, low-grade systemic coagulation and fibrinolytic activation are associated with androgen and insulin levels in polycystic ovary syndrome Arch Gynecol Obstet 2009 280 187 193 10.1007/s00404-008-0884-0 19107500

[B33] Goldberg RB Cytokine and cytokine-like inflammation markers, endothelial dysfunction, and imbalanced coagulation in development of diabetes and its complications JClin Endocrinol Metab 2009 94 3171 3182 10.1210/jc.2008-2534 19509100

[B34] Bobbert P Eisenreich A Weithäuser A Schultheiss HP Rauch U Leptin and resistin induce increased procoagulability in diabetes mellitus Cytokine 2011 56 332 337 10.1016/j.cyto.2011.05.019 21733717

[B35] Fang WQ Zhang Q Peng YB Chen M Lin XP Wu JH Cai CH Mei YF Jin H Resistin level is positively correlated with thrombotic complications in Southern Chinese metabolic syndrome patients J Endocrinol Invest 2011 34 e36 e42 20671416 10.1007/BF03347059

